# Endothelial Dysfunction and Brachial Intima-Media Thickness: Long Term Cardiovascular Risk with Claudication Related to Peripheral Arterial Disease: A Prospective Analysis

**DOI:** 10.1371/journal.pone.0093357

**Published:** 2014-04-16

**Authors:** Franz Hafner, Andrea Kieninger, Andreas Meinitzer, Thomas Gary, Harald Froehlich, Elke Haas, Gerald Hackl, Philipp Eller, Marianne Brodmann, Gerald Seinost

**Affiliations:** 1 Department of Internal Medicine, Division of Angiology, Medical University of Graz, Graz, Austria; 2 Clinical Institute of Medical and Chemical Laboratory Diagnostics, Medical University of Graz, Graz, Austria; VCU, United States of America

## Abstract

**Objective:**

Endothelial dysfunction plays a key role in the development, progression, and clinical manifestation of atherosclerosis, and in symptomatic peripheral arterial disease, endothelial dysfunction and enlarged intima-media thickness might be associated with increased cardiovascular risk. Flow-mediated dilatation and serologic parameters are used to evaluate individual endothelial function. Brachial intima-media thickness, a less recognized parameter of cardiovascular risk, is independently associated with coronary artery disease. The aim of this study was to evaluate the prognostic value of ultrasound and serologic parameters of endothelial function in relation to cardiovascular mortality in peripheral arterial disease.

**Design:**

monocentric, prospective cohort study.

**Methods:**

Flow mediated dilatation and brachial intima-media thickness were assessed in 184 (124 male) patients with peripheral arterial disease (Rutherford stages 2–3). Serologic parameters of endothelial function included asymmetric dimethylarginine (ADMA), symmetric dimethylarginine (SDMA), and L-homoarginine. Cardiovascular events were recorded during a follow-up of 99.1±11.1 months. Subjects who died of noncardiovascular causes were excluded from further analysis.

**Results:**

Eighty-two patients (44.6%) died during follow-up after a mean duration of 49.7±28.3 months. There were 49 cardiovascular deaths (59.8%) and 33 other deaths (40.2%). Flow mediated dilatation was associated with cardiovascular death [1.17% (0.0, 4.3) vs. 4.1% (1.2, 6.4), p<0.001]. Intima-media thickness was greater in patients who succumbed to cardiovascular disease [0.37 mm (0.30, 0.41)] than in survivors [0.21 mm (0.15, 0.38), p<0.001]. Brachial intima-media thickness above 0.345 mm was most predictive of cardiovascular death, with sensitivity and specificity values of 0.714 and 0.657, respectively (p<0.001). Furthermore, ADMA levels above 0.745 µmol/l and SDMA levels above 0.825 µmol/l were significantly associated with cardiovascular death (p<0.001 and 0.030).

**Conclusion:**

In symptomatic peripheral arterial disease, decreased flow mediated dilatation, enlarged intima-media thickness, and elevated levels of ADMA and SDMA were associated with increased cardiovascular risk.

## Introduction

Peripheral arterial disease (PAD) is a major health care burden [1]. PAD, as the manifestation of atherosclerosis in peripheral arteries, generally occurs in elderly patients and is commonly related to a variety of cardiovascular risk factors. In patients with peripheral arterial disease the ramifications of atherosclerosis are not limited to peripheral arteries. Patients with symptomatic and asymptomatic PAD are at heightened risk for myocardial infarction, stroke, and cardiovascular and all-cause mortality [2], [3].

Endothelial dysfunction plays a key role in development, progression and clinical manifestation of atherosclerosis. Brachial flow-mediated dilatation (FMD) is a validated, non-invasive ultrasound technique that measures endothelial dysfunction to predict cardiovascular risk [4], [5], [6], [7]. FMD determines the change in brachial artery diameter as a response to a blood flow stimulus after ischemic provocation. Shear stress at the arterial wall constitutes the established stimulus for FMD. As endothelial cells are equipped with mechanosensors to detect this force, vascular tone is modulated by a release of endothelial vasodilating mediators, including nitric oxide (NO), prostacyclin and endothelial hyperpolarizing factor [8]. NO drives the primary mechanism for FMD in healthy individuals, but the importance of other vasodilating agents like prostacyclin and endothelial hyperpolarizing factor may vary substantially in disease.

Asymmetrical dimethylarginine (ADMA) and its structural isomer symmetrical dimethylarginine (SDMA) are endogenous products of protein turnover that may be independent cardiovascular risk factors [9], [10]. Both molecules are released from proteins that have been post-translationally methylated and subsequently hydrolyzed. These proteins are involved in RNA processing and transcriptional control. Several cell types including endothelial cells elaborate ADMA, which directly inhibits the synthesis of nitric oxide (NO) by competitive binding to NO synthases. Both ADMA and SDMA may further reduce NO synthesis indirectly, inhibiting the cellular uptake of the NO precursor L-arginine [11]. ADMA has been shown to be a marker of endothelial dysfunction in humans [12]. The association of ADMA with all-cause mortality has been consistently demonstrated in various clinical settings. In ambulatory patients with peripheral arterial disease, ADMA but not SDMA seemed to be an independent risk factor for death [12]. In contrast to these data, serum concentrations of SDMA were independently associated with cardiovascular and all-cause mortality in a vascular risk population with coronary artery disease [9], [10]. Homoarginine, a cationic amino acid, may increase the availability of NO [13]. The serum level of homoarginine was reported to be an additional independent risk factor for all-cause mortality and cardiovascular death in patients undergoing coronary angiography [14]. Since the association between homoarginine levels and cardiovascular death has not been investigated in PAD, we aimed to determine the predictive value of established risk factors like ADMA and less recognized parameters like SDMA and homoarginine for cardiovascular death in PAD.

Intima-media thickness (IMT) is another non-invasive measurement used in cardiovascular disease. In contrast to the functional disturbance measured by FMD, IMT shows structural changes in the arterial wall even with subclinical atherosclerosis. Decreased FMD is further associated with progression of IMT in early atherosclerotic disease [15]. Carotid intima-media thickness (C-IMT) is known to be an independent cardiovascular risk factor [16]. Clinical studies revealed a weak correlation between brachial intima-media thickness (B-IMT) and carotid intima-media thickness [17], [18]. However, B-IMT was evaluated as an independent cardiovascular risk factor in elderly patients [19] and was further independently associated with the presence of coronary artery disease [18]. We previously reported that enlarged B-IMT was associated with restenosis after angioplasty of peripheral arteries [20]. The prognostic value of B-IMT regarding subsequent cardiovascular events in patients with symptomatic peripheral arterial disease has not yet been investigated.

Peripheral arterial disease is associated with an increased cardiovascular risk. We aimed to determine the additive prognostic value of serologic (ADMA, SDMA, homoarginine) and ultrasound parameters (FMD, B-IMT) on cardiovascular morbidity and mortality of symptomatic PAD patients presenting with claudication.

## Methods

### Study protocol

Between March 2002 and November 2004, consecutive patients undergoing their first endovascular revascularization of iliac and/or femoropopliteal arteries for intermittent claudication (Rutherford classification stage 2–3) were screened for inclusion in the study. Patients with acute myocardial infarction, unstable angina or stroke were excluded. Finally 184 consecutive patients (124 male, 60 female) gave written informed consent and participated in this prospective trial. Baseline data were collected on the day of the endovascular revascularization; four follow-up visits after 1, 3, 6 and 12 months were scheduled, as previously reported [20]. Between October 2010 and May 2011 all patients were contacted by the investigators to document the occurrence of cardiovascular events.

### Follow-up procedure and assessment of cardiovascular events

All patients received anti-platelet therapy with acetylsalicylic acid unless other medication was indicated for coronary artery disease. The patients' concomitant medication and the occurrence of cardiovascular events were noted at each study visit. Finally, all patients were contacted between October 2010 and May 2011 to answer a standard questionnaire covering PAD symptoms, interim medical history and concomitant medication. If the investigators could not reach a participant, the patient's general practitioner was contacted to provide the information, which included cardiovascular endpoints such as death, cause of death, stroke, myocardial infarction, and amputation. We recorded current medication and especially the use of anti-platelet therapies, coumarins, statins and the antihypertensive medication. The assessment of events was complemented and validated by a review of pertinent outpatient and inpatient data from all Styrian state hospitals, emergency services and pathology departments.

### Endothelial function and brachial intima-media thickness

All FMD measurements were made by the same trained technician according to standard guidelines [21]. Vasodilation of the brachial artery was measured with a linear array transducer, 8–13 MHz (Sequoia 512, ACUSON Corp., Charleston Rd., Mountain View, CA, USA).

After a 5-minute rest in the supine position in a climate-controlled room, the brachial artery was examined in a longitudinal plane between 1 and 5 centimetres above the antecubital fossa by continuous grey scale imaging. The position of the transducer was marked on the skin to ensure that the following measurements were taken from the same vascular segment. The end-diastolic distance between the two intimal interfaces was measured with ECG gating during image acquisition in a one-centimetre-long segment at least three times; a mean of these values was calculated for subsequent analysis. All the diameters were taken in the end-diastolic phase, which was defined as the beginning of the R-wave in the ECG. A blood pressure cuff on the forearm was inflated for 5 minutes with a constant pressure 50 mmHg above the systolic brachial blood pressure. The post-ischemic brachial arterial diameter was measured 45 seconds after cuff release. FMD was defined as the change in post-ischemic diameter as a percentage of the baseline diameter.

**Figure 1:**
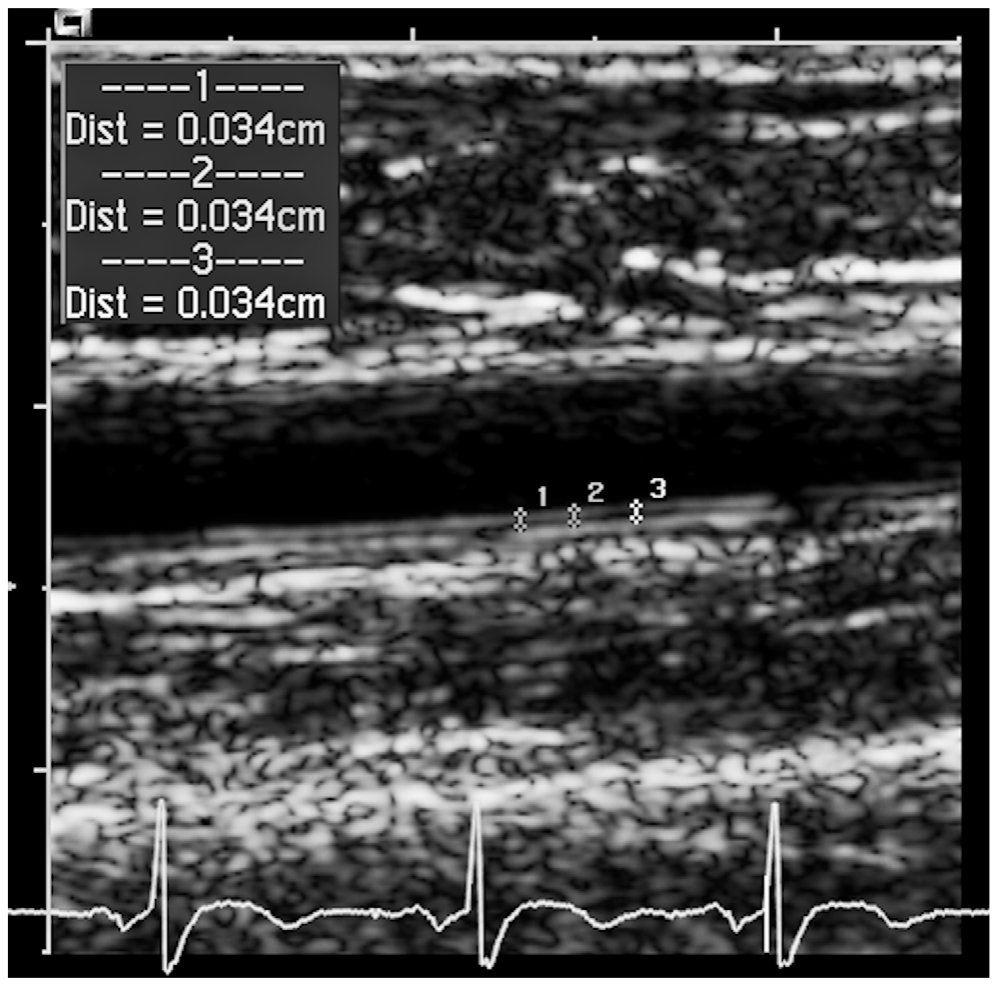
Grey scale ultrasound image of the right brachial artery of a 74-year-old female patient. Brachial IMT is measured three times in a one-centimeter-long segment. ECG triggering indicates the measurement in the enddiastolic phase.

Flow-independent vasodilatation was evaluated by nitroglycerin-mediated dilatation (NMD). At least 15 minutes after the FMD procedure the brachial arterial diameter was measured before and 3 minutes after sublingual administration of nitroglycerin (NTG 0.3 mg spray) [22]. We defined brachial intima-media thickness (B-IMT) as the mean of at least three different measurements of IMT in a one-centimeter-long segment of the brachial artery above the antecubital fossa. Brachial IMT was measured on the far wall in the end-diastolic phase before cuff inflation for the FMD test as presented in figure 1 [23].

### Biochemical analyses

Fasting blood samples were drawn at the baseline visit; the centrifugated serum was stored at −70°C until further analysis in March 2011. According to previous reports, the investigated biomarkers can be assumed to be stable. [24]. We measured SDMA, ADMA and homoarginine by high-performance liquid chromatography (HPLC) with the solid phase extraction and precolumn derivatization technique first described by Teerlink [25], with slight modifications [26]. Within-day CVs (coefficients of variation) for SDMA were 4.6% (0.60 µmol/L) and 1.9% (1.0 µmol/L), and between-day CVs were 9.8% (0.60 µmol/L) and 6.1% (1.0 µmol/L). Within-day CVs for ADMA were 3.1% (0.62 µmol/L) and 1.0% (2.0 µmol/L), and between-day CVs were 9.0% (0.62 µmol/L) and 2.2% (2.0 µmol/L). Within-day CVs for homoarginine were 4.7% (1.21 µmol/L) and 2.2% (3.53 µmol/L), and between-day CVs were 7.9% (1.25 µmol/L) and 6.8% (3.66 µmol/L).

### Statistical analysis

Parametric data are given as mean ± SD, unless otherwise indicated. Student's t test and chi squared analysis were used to compare mean value and frequency between two groups in the case of normally distributed values, which were confirmed by the Kolmogorov-Smirnov test. With skewed data like intima-media thickness, FMD at baseline, homoarginine, ADMA, and SDMA, median values with interquartile ranges are presented. Demographics were calculated for cardiovascular death, noncardiovascular death and survivors by ANOVA analysis and Kruskal Wallis test if indicated. In a second step noncardiovascular deaths were excluded from further analysis. Optimal cut-off values for the parameters FMD, NMD, B-IMT, homoarginine, ADMA and SDMA as potential predictors of subsequent cardiovascular death were evaluated by ROC analysis. The survival analysis was completed by Cox regression statistics evaluating these cut-off values after adjustment for age, gender, creatinine clearance, smoking behaviour, and hypertension. Statistical significance was assumed for p values<0.05. Fisher's exact test was used as a chi-squared test to compare frequencies of a condition between two groups. Factors associated with cardiovascular death were combined and the Jonckheere-Terpstra test was used for trend statistics related to cardiovascular death. Statistics were assessed with IBM SPSS version 20.0.

### Ethics Statement

The study was approved by the Institutional Review Board of the Medical University Graz, Austria (EK 23-038 ex 10/11). Signed written informed consent was obtained for all study related procedures.

## Results

### Clinical characteristics and cardiovascular risk profile

In all, 184 patients (124 male and 60 female) participated in this trial. Three patients withdrew their informed consent to repeated assessment of FMD, but finally agreed to provide information on cardiovascular events. As expected for a study population suffering from PAD, mortality was as high as 44.6% in the follow-up period of 99.1±11.1 months. All cause deaths accounted for 82 patients (44.6%), 49.7±28.3 months after endovascular treatment. Cardiovascular causes of death predominated in our study population, accounting for 49 patients (59.8%), whereas the remaining 33 deaths (40.2%) were assigned to cancer (22 cases), infectious diseases (5 cases), trauma (2 cases), liver cirrhosis (2 cases) and respiratory failure (2 cases). These 33 patients with nonvascular causes of death were excluded from further analysis, so that the final study population eligible for statistical calculation comprised 151 patients (101 male) with symptomatic PAD. Differences in age, prevalence of hypertension, smoking status, and biochemical parameters between survivors and non-survivors are presented in Table 1. Since participants were enrolled over a period of two years, the length of follow-up varied, but a correction for this variation did not influence the results.

**Table 1:** Concomitant associated diseases and medication at baseline.

	Survivor (n = 102)	Cardiovascular Death (n = 49)	Total (n = 151)	Sign.
Age (years)	64.3±10.2	72.3±9.3	66.9±10.6	**<0.001**
BMI	26.9±3.0	26.3±3.3	26.7±3.1	0.318
Male gender *n* (%)	73 (71.6)	28 (57.1)	101 (66.9)	0.097
Hypertension *n* (%)	76 (74.5)	46 (93.9)	122 (80.8)	**0.004**
Smoker *n* (%)	80 (78.4)	26 (53.1)	106 (70.2)	**0.002**
Current smoker *n* (%)	42 (41.2)	12 (24.5)	54 (35.8)	0.048
Diabetes mellitus *n* (%)	27 (26.5)	22 (45.8)	49 (32.7)	0.025
Hyperlipidemia *n* (%)	54 (52.9)	24 (49)	78 (51.7)	0.729
Hemoglobin (g/dl)	14.2±1.5	13.0±1.7	13.8±1.6	**<0.001**
Homocysteine (µmol/l)	15.0±4.8	16.9±4.6	15.5±4.8	0.048
Total cholesterol (mg/dl)	208.2±47.5	196.7±51.5	204.6±48.9	0.196
HDL cholesterol (mg/dl)	50.8±14.6	49.6±15.2	50.5±14.8	0.650
LDL cholesterol (mg/dl)	124.4±39.4	113.9±38.1	121.2±39.2	0.149
Lp (a) (mg/dl)	13.1 (9.5, 50.5)	18.6 (10.0, 45.7)	13.3 (9.5, 47.6)	0.310
High sensitive CRP (mg/l)	4.0 (2.0, 8.8)	5.5 (2.9, 8.8)	4.2 (2.2, 8.8)	0.140
Creatinine Clearance (ml/min/1.7)	70.6±23.5	56.9±26.2	66.1±25.1	**0.002**
HbA1c (%)	5.7 (5.4, 6.3)	6.1 (5.5, 6.9)	5.8 (5.4, 6.5)	**0.046**
Cerebrovascular disease *n* (%)	52 (51.0)	23 (46.9)	92 (50.0)	0.881
Coronary artery disease *n* (%)	20 (19.6)	12 (24.5)	39 (21.2)	0.790
Statin *n* (%)	49 (48.0)	24 (49.0)	86 (46.7)	0.643
Aspirin *n* (%)	75 (73.5)	31 (63.3)	128 (69.6)	0.405
Clopidogrel *n* (%)	8 (7.8)	6 (12.2)	17 (9.2)	0.682
VKA *n* (%)	7 (6.9)	9 (18.4)	19 (10.3)	0.091
Antihypertensive drug *n* (%)	69 (67.6)	43 (87.8)	129 (70.1)	**0.001**

BMI Body Mass Index; HDL High density lipoprotein; LDL Low density lipoprotein; Lp (a) Lipoprotein (a); HbA1c Hemoglobin A1c; VKA Vitamin K antagonists.

### Endothelial function and CV death

Next, we compared markers of endothelial function in survivors and non-survivors who succumbed to cardiovascular events. We observed lower FMD values in the group non-survivors than in the survivors, with values of 1.17% (0.0, 4.3) vs. 4.1% [(1.2, 6.4), p<0.001], as presented in Table 2. Mean brachial IMT was greater in non-survivors, with a value of 0.37 mm (0.30, 0.41) as compared to survivors [0.21 mm (0.15, 0.38), p<0.001). Regarding our serologic parameters of vascular function, homoarginine was slightly decreased in non-survivors with a value of 1.43 µmol/l (1.00, 1.84) as opposed to 1.63 µmol/l (1.24, 2.07), p = 0.050. Both ADMA and SDMA were higher in the non-survivors than in the survivors, with mean values of 0.75 µmol/l (0.68, 0.86) vs. 0.71 µmol/l [(0.66, 0.75), p = 0.006] and 0.85 µmol/l (0.67, 1.02) vs. 0.69 µmol/l [(0.60, 0.80), p<0.001] respectively.

**Table 2:** Endothelial function, brachial intima-media thickness, ankle-brachial index and serologic parameters of endothelial function in the different subgroups of patients with cardiovascular death events and in survivors.

	Death vascular (n = 49)	Survivor (n = 102)	Total (n = 151)	Sign.
FMD (%)	1.17 (0.0, 4.3)	4.1 (1.2, 6.4)	3.0 (0.3, 6.0)	<0.001
NMD (%)	12.32±7.04	14.74±7.57	13.98±7.47	0.087
B-IMT (mm)	0.37 (0.30, 0.42)	0.21 (0.15, 0.38)	0.30 (0.16, 0.40)	**<0.001**
ABI	0.64 (0.51, 0.88)	0.71 (0.59, 0.89)	0.69 (0.56, 0.89)	0.115
Homoarginine (µmol/l)	1.43 (1.00, 1.84)	1.63 (1.24, 2.07)	1.58 (1.13, 1.99)	0.050
ADMA (µmol/l)	0.75 (0.68, 0.86)	0.71 (0.66, 0.75)	0.72 (0.67, 0.77)	**0.006**
SDMA (µmol/l)	0.85 (0.67, 1.02)	0.69 (0.60, 0.80)	0.70 (0.62, 0.90)	**<0.001**

FMD Flow Mediated Dilatation; NMD Nitrogen Mediated Dilatation; B-IMT Brachial Intima-Media Thickness; ABI Ankle-Brachial Index; ADMA Asymmetric Dimethylarginine; SDMA Symmetric Dimethylarginine.

**Figure 2:**
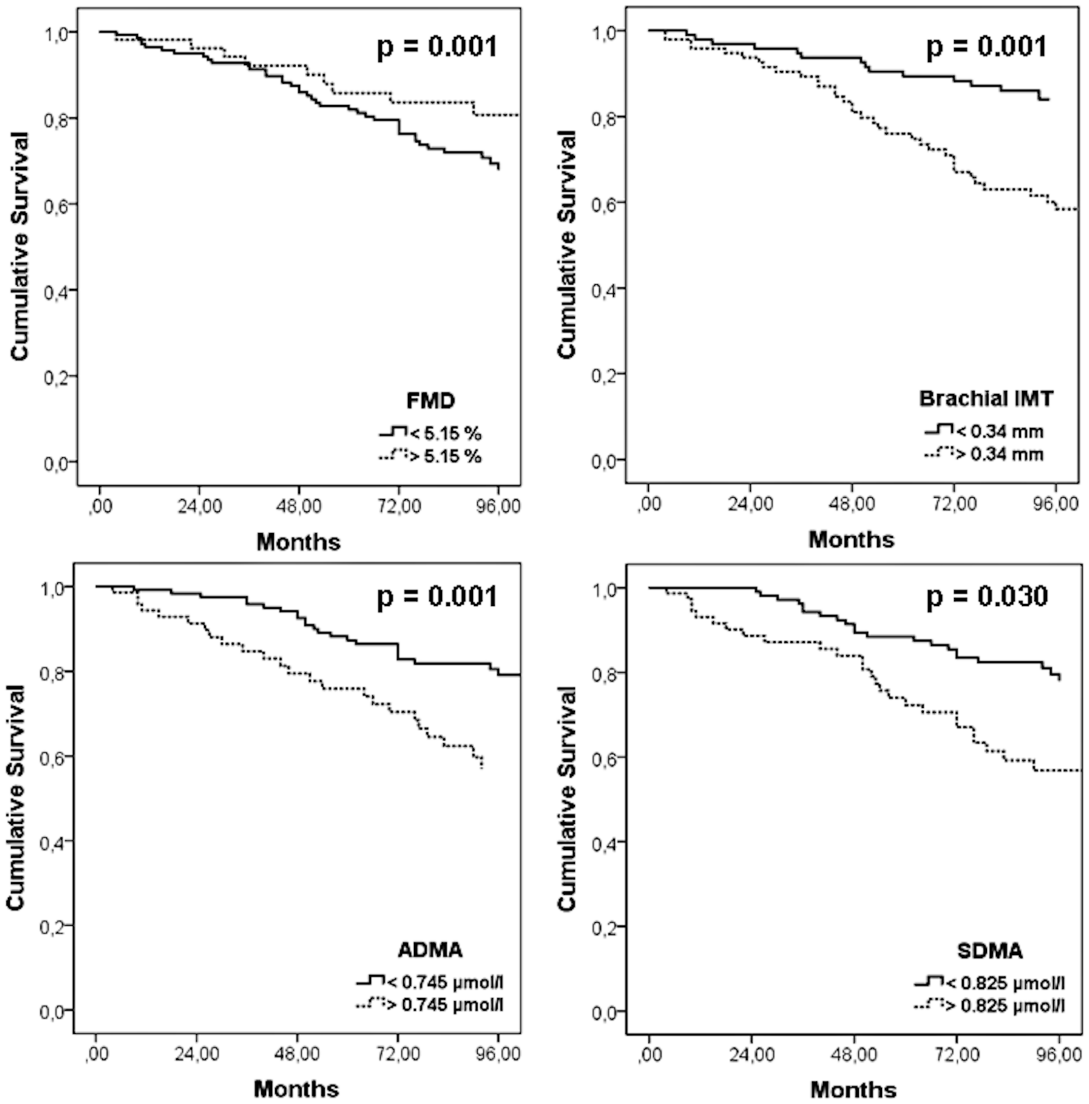
Cox regression survival plots showing cardiovascular mortality as a function of FMD, B-IMT, ADMA and SDMA with adjustment for age, gender, arterial hypertension, smoking behavior, and creatinine clearance.

ROC analysis evaluated optimal cut-off values for the above mentioned vascular parameters, differentiating survivors from non-survivors. The ROC analysis achieved statistical significance for the following cut-off values: FMD (5.15%, p = 0.002), B-IMT (0.034 mm, p<0.001), ADMA (0.745 µmol/l, p = 0.002) and SDMA (0.825 µmol/l, p = 0.001), as presented in Table 3. Mean survival time (95% CI) differed depending on these cut-off values, as presented in Table 4 and Figure 2. Summarizing the prevalence of FMD below the cut-off, and B-IMT, ADMA and SDMA above the mentioned cut-off values, we observed no cardiovascular death in patients without one of these cardiovascular risk factors, whereas the frequency increased to 10.0%, 40.4%, 56.0%, and 81.8% if one, two, three or all four of these factors were above the cut-off value (p<0.001), as presented in Table 5.

**Table 3:** ROC analyses evaluating cut-off values for flow mediated dilatation (FMD), nitroglycerin-mediated dilatation (NMD), brachial intima-media thickness (B-IMT) and serologic parameters of endothelial function predicting cardiovascular death.

	Area ± SE	Asymp Sign.	95% CI	Cut-off	Sensitivity	Specifity
FMD	0.325±0.054	0.002	0.219–0.430	5.15%	0.204	0.204
NMD	0.423±0.054	0.164	0.317–0.528	15.69%	0.390	0.567
B-IMT	0.730±0.046	**<0.001**	0.641–0.820	0.345 mm	0.714	0.657
Homoarginine	0.393±0.055	0.055	0.286–0.501	1.53 µmol/l	0.500	0.474
ADMA	0.675±0.055	**0.002**	0.567–0.783	0.745 µmol/l	0.500	0.732
SDMA	0.692±0.053	**0.001**	0.588–0.796	0.825 µmol/l	0.563	0.814

**Table 4:** Cox regression survival analysis for cut-off values of flow-mediated dilatation (FMD), brachial intima-media thickness (B-IMT), ADMA and SDMA, all parameters adjusted for age, gender, arterial hypertension, eGFR, and smoking behavior.

Parameter	Group	Number of cases	Number of events	Number censored	Mean survival time in months (95% CI)	Sign.
FMD (%)	<5.15	108	39	69 (63.9)	97 (90, 105)	0.001
	>5.15	43	10	33 (76.7)	121 (108, 134)	
IMT (mm)	<0.345	80	14	66 (82.5)	111 (104, 118)	0.001
	>0.345	70	35	35 (50.0)	97 (85, 108)	
ADMA (µmol/l)	<0.745	94	24	70 (74.5)	119 (111, 127)	0.001
	>0.745	50	24	26 (52.0)	80 (70, 91)	
SDMA (µmol/l)	<0.825	99	21	78 (78.8)	110 (104, 116)	0.030
	>0.825	45	27	18 (40)	86 (71, 101)	

**Table 5:** Additive risk factor analysis of flow mediated dilatation (FMD), brachial intima-media thickness (B-IMT), ADMA and SDMA for cardiovascular mortality.

Factors above cut-off value	CV death (n, % within group)	Survivors (n, %)	Total (n, % of study group)	Significance
0	0 (0%)	13 (100)	13 (8.6%%)	
1	5 (10.0%)	45 (90%)	50 (33.1)	
2	21 (40.4%)	31 (59.6%)	52 (34.4%)	
3	14 (56.0%)	11 (44.0%)	25 (16.6%)	
4	9 (81.8%)	2 (18.2%)	11 (7.3%)	
Total	49 (32.5%)	102 (67.5%)	151 (100%)	<0.001

Factors: FMD<5.15%, IMT>0.34 mm, ADMA>0.745 µmol/l, SDMA >0.825 µmol/l; 0: no risk factor positive; 1: one risk factor positive; 2: two risk factors positive; 3: three factors positive, 4: all four factors positive.

## Discussion

The following main findings emerged from our study: (1) Cardiovascular events represent the most frequent reason for death in peripheral arterial disease. (2) Endothelial dysfunction, measured as decreased FMD response, was associated with cardiovascular death in patients with claudication related to PAD. (3) The combination of decreased FMD and increased values of B-IMT, ADMA and SDMA was further significantly associated with cardiovascular death in peripheral arterial disease.

The prevalence of PAD is rapidly growing in our aging community while endovascular therapies have increased therapeutic strategies [27]; however, these invasive approaches usually focus on the local problem of an obstructed peripheral artery. Because atherosclerosis is not limited to the extremities, physicians should be alert for cardiovascular complications and events in PAD patients. A large prospective study showed that patients with PAD had a significantly increased cardiovascular risk compared to patients without PAD [2]. The present prospective observational study of symptomatic PAD patients with intermittent claudication showed all-cause mortality of 44.6% after 49.7±28.3 months, whereby the majority (59.8%) of these deaths were due to cardiovascular events. These findings are in line with previous studies in this field [28], [29] and patients with symptomatic PAD do have a 10- to 15-fold increased risk of death compared to age-matched controls [30].

Endothelial dysfunction, as measured by decreased FMD, was recently studied by Allan et al. in PAD patients as compared to healthy subjects [31], showing that patients with PAD had a significantly lower FMD than healthy subjects. We also saw significantly decreased FMD values among non-survivors [1.17% (0.0, 4.3)] as compared to survivors 4.1% [(1.2, 6.4), p<0.001]. Furthermore, decreased FMD response was associated with non-fatal myocardial infarction. The FMD value of 3.0% (0.3, 6.0) in our study group was much lower than the reported FMD values of elderly patients in many similar studies, which may reflect advanced endothelial dysfunction in patients with symptomatic PAD. FMD response was investigated 45 seconds after cuff deflation, following the guidelines of the International Brachial Artery Reactivity Task Force [21]. Our approach so may underestimate the maximum flow response compared to continuous recording of the dilation curve [32]. In contrast to this endothelial dependent measurement, we observed no statistical difference in endothelial independent NMD values in the group with CV death compared to survivors (12.32±7.04% vs. 14.74±7.57%).

Elevated values of B-IMT are associated with the atherosclerotic burden in coronary artery disease [18], [33], but their relevance in PAD has not yet been thoroughly investigated. We observed greater B-IMT in patients with cardiovascular death [0.37 mm (0.30, 0.42)] than in survivors [0.21 mm (0.15, 0.38)], and in patients with non-fatal myocardial infarction [0.35 mm (0.21, 0.45)] than in event-free survivors [0.29 mm (0.15, 0.40)]. We previously reported measurement of B-IMT as a simple and reliable tool for identifying patients at risk for re-stenosis after endovascular treatment in PAD [20]. Carotid and brachial IMT are both influenced by the individual cardiovascular risk profile in this patient group [17], whereby greater B-IMT reflects progressive atherosclerotic disease. In addition to FMD, B-IMT may also be used as a tool to assess cardiovascular risk in patients with cardiovascular risk factors or apparently advanced atherosclerosis.

Homoarginine, ADMA and SDMA were evaluated as predictive endothelial markers of cardiovascular risk in PAD patients. Homoarginine increases the availability of NO and low concentrations of homoarginine are associated with endothelial dysfunction. L-homoarginine was found to significantly correlate with brachial artery diameter and endothelium-dependent brachial artery FMD [34]. However, in our cohort homoarginine did not differ significantly between survivors and non-survivors. As the relatively small sample size may be an explanation for the lack of correlation of homoarginine and CV death in our study group, we excluded this parameter from the survival analysis.

Conversely, ADMA and SDMA were elevated in non-survivors compared to survivors. This finding goes along with a previous report of Böger et al. describing the predictive value of elevated ADMA plasma levels for mortality and major cardiovascular events in an unselected cohort of symptomatic and asymptomatic PAD patients [35]. The authors reported an association between increased SDMA plasma levels and mortality, which was explained by a relation between SDMA and renal impairment. Patients with advanced renal impairment (i.e. GFR below 30 ml/min) were excluded from our study because of the risk of contrast agent induced nephropathy. ADMA and SDMA concentrations were significantly associated with cardiovascular death in our cohort of symptomatic PAD patients in Rutherford stages 2 and 3. Since ADMA is a competitive inhibitor of NOS, elevated ADMA concentrations may reduce NO generation, which in turn may subsequently evoke endothelial dysfunction [12]. Our data confirm the principal finding in previous studies of elevated ADMA and SDMA levels in vascular diseases like PAD.

The aim of our study was to evaluate different structural and serological parameters of endothelial function and the additive value of B-IMT for calculation of cardiovascular risk in symptomatic PAD. Decreased FMD and increased levels of B-IMT, ADMA and SDMA were indeed associated with cardiovascular death. The strongest correlation was observed for SDMA and B-IMT with respective values of 0.383 and 0.327, whereas ADMA and FMD were less predictive. We finally combined these four parameters and assessed survival trends statistically. The combination of FMD, B-IMT, ADMA and SDMA turned out to be significantly associated with cardiovascular death in PAD. In contrast, we did not observe a correlation between a reduced ankle-brachial index and cardiovascular death. This finding might be related to the inclusion of a homogeneous cohort of PAD patients suffering from claudication. Since asymptomatic subjects and patients with critical limb ischemia were excluded, a reduced variation of the ankle-brachial index was to be expected.

### Limitations

The strength of our study is the homogenous cohort of symptomatic PAD patients in Rutherford stages 2–3 and their long term follow-up. Our study has several limitations: First, we are not able to provide hemodynamic data such as ABI during the follow-up period. Second, we excluded asymptomatic PAD patients and patients with resting pain or ischemic skin lesions (Rutherford 4–6) from our study. Our conclusions therefore refer only to patients with claudication, but morbidity and mortality in patients with chronic critical limb ischemia are known to be even worse. Since measurement of brachial IMT is less established and technically more challenging than carotid IMT, we cannot say anything conclusive about its reliability. Our data nonetheless encourage us to include this parameter in cardiovascular risk assessment. Finally, investigation of FMD response 45 seconds after cuff deflation may underestimate the maximum flow response when compared to a continuous recording of the dilation curve [32]. This issue deserves further attention.

In all, this prospective study revealed a statistical association between structural changes of the brachial artery measured by brachial IMT and cardiovascular death. Based on our evidence that elevated serologic parameters of endothelial function like ADMA and SDMA are significantly associated with elevated cardiovascular risk, FMD, B-IMT, ADMA and SDMA nicely augment our armamentarium for identifying patients at high cardiovascular risk and therefore should be integrated in future cardiovascular risk management of PAD patients. Interventional studies should be designed to determine whether intensified therapeutic strategies in the high risk group can translate into improved clinical outcome.
